# Supplementary material for: A whole-animal phenotypic drug screen identifies suppressors of atherogenic lipoproteins
Source: eLife. 2026 Aug 27;14:RP105314. doi: 10.7554/eLife.105314 (PMC13521566; doi:10.7554/eLife.105314)
Supplement: Supplementary file 2 [file elife-105314-code1.zip › JHDL_primary_result/Polysorbate┬á65/Polysorbate┬á65_JHDL_32_035_020_2020_01_24_10_41.pdf]

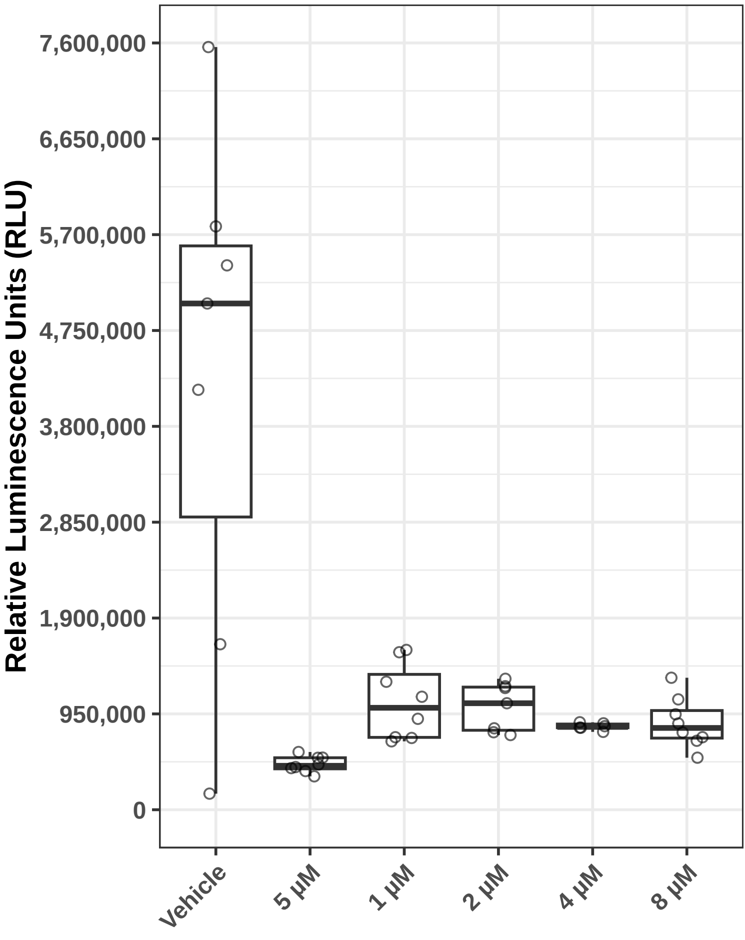
Relative Luminescence Units (RLU)
7,600,000
6,650,000
5,700,000
4,750,000
3,800,000
2,850,000
1,900,000
950,000
0
Vehicle
5 µM
1 µM
2 µM
4 µM
8 µM
